# Treatment as Prevention: unanswered questions and progress to date

**DOI:** 10.7448/IAS.17.4.19521

**Published:** 2014-11-02

**Authors:** Stefano Vella

**Affiliations:** Department of Therapeutic Research and Medicines E, Istituto Superiore di Sanita’, Rome, Italy

## Abstract

Prevention of HIV infection has recently acquired new effective tools, based on the provision of antiretrovirals, to both decrease the “source” of HIV infection and/or to decrease host susceptibility to HIV infection. The milestone concept of using antiretrovirals for prevention has been tested already in the 1990s, to interrupt maternal–child. The suggestion of using antiretrovirals to decrease inter-human HIV transmission was born already in 2006, and definitely proven by a randomized controlled study on HIV-discordant couples demonstrating an astonishing 96% efficacy, and with numerous studies proving the efficacy of pre-exposure prophylaxis. It now appears clear that antiretroviral therapy not only provides clinical benefit to the individual (although the exact starting time remains controversial, in terms of risk-benefit ratio and public health policy) but has the potential of decreasing the incidence of new infections at a population level. The concept of Treatment as Prevention is now gaining momentum, as a way to progressively end the AIDS epidemic by expanding the access to antiretrovirals, with “test and treat” being the ultimate possibility, although not already tested in the field and with huge implementation barriers. The presentation will deal with successes, failures, and foreseen barriers of using antiretrovirals for prevention, at the individual and population level, also including the social issues, the need to target key-affected populations, and, finally, the perspectives of new technologies under development.

